# Why Some Adolescents Are Open To Their Parents’ Political Communication

**DOI:** 10.1007/s10964-022-01653-x

**Published:** 2022-07-08

**Authors:** Håkan Stattin, Katharina Eckstein, Erik Amnå

**Affiliations:** 1 Department of Psychology, Uppsala University, Box 1225, SE-75142 Uppsala, Sweden; 2 Department of Educational Psychology, Friedrich Schiller University of Jena, Humboldtstr. 27, 07743 Jena, Germany; 3 School of Humanities, Education and Social Sciences, Örebro University, SE-70182 Örebro, Sweden

**Keywords:** Political socialization, Perceptual accuracy, Parents, Adolescents, Political role model, Political sophistication

## Abstract

This study examines the conditions that make adolescents open to their parents’ attempts at political socialization. Based on a reformulation of the perceptual accuracy argument, that parents’ messages are filtered through correct perceptions of these messages by adolescents, the study suggests that adolescents who accurately recognize their parents’ high political sophistication are particularly likely to attend to and be open to their parents’ political communication. This proposition was tested using cluster analysis of a sample of 505 Swedish upper-secondary students and their parents (51% girls; *M*age = 16.56, *SD* = 0.67). The analysis yielded two clusters where adolescents correctly identified (26%) and failed to correctly identify (22%) their parents’ high political sophistication, and three clusters where both parents and adolescents reported low or medium parental political sophistication (10%, 11%, and 32%). In confirmation of the hypothesis, members of the cluster group of adolescents who correctly recognized their parents’ high political sophistication were particularly aware of parents’ political socialization attempts and receptive to parents’ political communication. Moreover, these youth considered their parents’ political views as important and, accordingly, seemed to perceive their parents as political role models.

## Introduction

Despite the classical belief that parents are the main political socialization agents of their children, the literature up to now shows that there is little correspondence between how parents and adolescents perceive their political interactions with each other (Jennings et al., 2009; Saphir & Chaffee, 2002; Stattin & Kim, 2018). This indicates that openness on the part of adolescents to their parents’ political socialization is not universal but might only be applicable in some cases. Applying a perceptual accuracy argument to parents’ political socialization, it is proposed that adolescents’ attention to their parents’ political communication is facilitated if they correctly recognize their parents’ high political interest and knowledge (i.e., parents are highly politically interested and knowledgeable, and adolescents perceive their parents to be highly politically interested and knowledgeable). It is primarily among these adolescents that a recognition of their parents’ political support, influence and an openness to their political communication can be expected. Further, the ability to recognize their parents as politically interested and knowledgeable requires some rudimentary political interest on part of the adolescents themselves. The present study examines the validity of these two propositions.

### Shared Values, Political Interest, and Political Discussions at Home

There is a lack of congruence between parents’ and adolescents’ sociopolitical values and attitudes, reports about political discussions, and perceptions of each other’s political interest. Excepting party identification, the congruities between adolescents’ and their parents’ sociopolitical views are typically moderate to low (Geißler, 1996; Gniewosz & Noack, 2006; Neundorf et al., 2013; Oswald & Schmid, 2006; Roest et al., 2009; Stattin & Kim, 2018). Parents’ and adolescents’ reports of how often they engage in political discussions with each other show either a low association (Meadowcroft, 1986; Ritchie & Fitzpatrick, 1990; Saphir & Chaffee, 2002; Tims & Masland, 1985), or, at most, a moderate association (Pacheco, 2008). Parents’ political interest shows only a small to moderate association with their children’s political interest (Jennings et al., 2009; Stattin et al., 2021). From the viewpoint that parents politically socialize their children through political discussions at home, these findings of low congruence in key domains—values and attitudes, political discussion at home, and political interest—may seem discouraging.

Stronger congruities are reported in studies based on adolescents’ perceptions. Some have found moderate to high associations between adolescents’ reports of their own values or views and their perceptions of their parents’ values or views (Acock & Bengtson, 1980; Gniewosz & Noack, 2006; Gniewosz et al., 2008; Ojeda & Hatemi, 2015). Others have found moderate to high associations between adolescents’ own political interest and their perceptions of how often political discussions with parents take place (Dostie-Goulet, 2009; Kim & Stattin, 2019; Russo & Stattin, 2017). Still other studies have compared adolescents’ perceptions of their parents’ values and political interactions with their parents’ views: adolescents tend to perceive their parents’ values as similar to their own (Acock & Bengtson, 1980), and, vice-versa, parents tend to perceive their children’s values as similar to their own (Stattin & Kim, 2018). Further, parents’ and adolescents’ perceptions of how frequently political discussions in the family take place are closely linked to their respective political interests (Stattin et al., 2021). These studies (Stattin & Kim, 2018; Stattin et al., 2021) also show that parents’ and adolescents’ values, as well as parents’ and adolescents’ reports on the amount of political discussion at home, are not well matched. Parents and adolescents seem to have little insight into each other’s perceptual worlds when it comes to politics.

### Intergenerational Transmission

Given that there is little evidence in the political socialization literature pointing to distinctive intergenerational similarities, the following question can be raised: Are there some types of families in which it can be expected that adolescents are more open to their parents’ political socialization than in other families? At heart, this question aims at specifying the conditions of a successful intergenerational transmission. Although early research did not provide strong evidence for sociopolitical intergenerational transmission (Jennings & Niemi, 1968), later follow-up studies re-examined the idea of family transmission. Jennings et al. (2009) proposed and found empirical support for the idea that, for parents to be influential socialization agents, they need to express their views consistently over time; otherwise, their children will not accurately perceive their political messages. This much-cited study gives perhaps the best explanation up to now for why young people come to share, or not share, their parents’ political ideologies, values, and attitudes.

In the present study, another explanation, which empathizes adolescents’ perceptual characteristics, is proposed. The idea behind it has empirical support in different literatures. Tedin (1974) suggested that children’s *perceptual accuracy* in recognizing their parents’ political values and attitudes may explain the success and failure of parents’ political socialization attempts. In value socialization theory, which pertains to developmental psychology, children need to correctly perceive their parents’ values and attitudes, and then accept these values and attitudes as their own, for internalization to take place (Grusec & Goodnow, 1994). More recently, in sociology, a similar type of two-step model has been advanced (Ojeda & Hatemi, 2015). In these models, decisions to accept parents’ values and attitudes depend on young people’s accurate perceptions. An analogous but broader-ranging perceptual accuracy argument is presented in the current study, namely that adolescents’ openness to parents’ political socialization attempts will depend on their perception of parents’ high political sophistication. It is hypothesized that the likelihood of adolescents’ openness to parents’ political socialization increases if adolescents correctly perceive their parents to be highly politically sophisticated. If adolescents do not perceive their parents’ high level of political sophistication, they are not likely to recognize parents’ political support and influence nor to be susceptible to parents’ political communication—regardless of the political values and attitudes the parents want to transfer. In the following, the term *political sophistication* is attached to parents who are perceived to be politically interested and knowledgeable by their adolescent children. These are characteristics of being politically well-informed that adolescents should be able to observe in their parents. In his classical article on political sophistication, Luskin (1990) defined political sophistication in terms of the roles played by people’s interest in politics and their exposure to political information in the media. This is in line with definitions reported in the current literature (Rapeli & von Schoultz, 2021; Vegetti & Mancuso, 2020). Although previous studies have shown that many adolescents have limited insight into their parents’ sociopolitical values, beliefs, and opinions, this study is particularly concerned with adolescents who make correct assessments of their parents’ high political sophistication and its consequences for political interactions at home.

The present study proposes another explanation for the mechanism behind family transmission than the one advanced by Jennings et al. (2009). The classical definition of political socialization in the family puts much emphasis on the social learning process through which adolescents acquire their political interests, values, ideologies, and opinions (Barrett & Brunton-Smith, 2014). Adolescents comply with and adopt their parents’ views through learning, observation, and modeling (Jennings et al., 2009). Accordingly, in their study, Jennings and colleagues measured the consistency over time in parents’ attitudes but they did not assess how the children perceived these attitudes. By contrast, the perceptual accuracy argument (Grusec & Goodnow, 1994; Ojeda & Hatemi, 2015; Tedin, 1974) suggests that parents’ messages and influences need to pass through the perceptions of adolescents. Applied to political socialization, if adolescents correctly perceive that their parents are highly politically sophisticated, then they are more likely both to recognize their parents’ socialization attempts and subsequently be more open to their parents’ political views than other adolescents. They will view their parents as role models on political and societal matters more than other adolescents. Further, adolescents who correctly identify their parents’ high political sophistication should be more likely to perceive that they have frequent political discussions with their parents than other adolescents. It is less likely that adolescents who fail to recognize their parents’ political sophistication will take part in, or even recall, such discussions. Adolescents’ awareness of having joint political discussions with parents is potentially a hallmark of the group characterized by correct perception of parents’ high political sophistication.

### Adolescents’ Political Interest

Why, though, do some adolescents correctly perceive the high political sophistication of their parents, whereas other adolescents, who also have highly politically sophisticated parents, do not? Political interest, defined as being attentive to political issues (Levy & Akiva, 2019), is one of the most potent predictors of diverse aspects of adolescents’ political attitudes, behaviors, and participation (Prior, 2019). Adolescents’ reports on political discussions with parents and parental support are intimately linked to their own political interest (Stattin et al., 2021). One reason why some adolescents accurately perceive the high political sophistication of their parents might be that they have greater political interest than other adolescents to start with. It is not to be expected that adolescents who are unaware of their parents’ high political sophistication are particularly interested in politics.

Altogether, whereas the learning position (Jennings et al., 2009) proposes that it is parents’ ability to consistently communicate their political values, beliefs, and opinions that affects their adolescents’ political development, the reformulated perceptual accuracy hypothesis suggests that it is particularly the adolescents who correctly perceive their parents’ high political sophistication who will recognize their parents’ socialization messages and be open to parents’ views. This ability to discern their parents’ high political sophistication should be strongly related to these adolescents’ own political interest.

## Current Study

Drawing on a sample of upper-secondary students from Sweden, this study seeks to answer the question whether there are some families in which adolescents are more open to their parents’ political socialization than in others. Hypothesis 1: It is proposed that adolescents’ accurate perception of their parents’ high political sophistication plays a key role for their openness to parents’ political socialization. More precisely, it is expected that—compared to others—adolescents who correctly perceive their parents’ high political sophistication are (1) more aware of parents’ political socialization attempts (i.e., recognize that parents try to make them aware about political and environmental issues, recognize that parents provide political information), (2) are more open to parents’ political communication (i.e., susceptible to parents’ political communication, consider parents’ political views important), and (3) perceive that they have frequent political discussions with their parents. On part of the parents, in turn, it is expected that, irrespective of whether their children are open to their political communication or not, parents with high political sophistication should try to politically influence their adolescent children (Jennings et al., 2009). Nevertheless, only the adolescents who correctly recognize their parents’ high political sophistication are assumed to recognize these political socialization attempts. Second, it is hypothesized that a main reason that adolescents are able to recognize their parents’ high political sophistication is their own level of political interest (Hypothesis 2).

Cluster analysis of adolescents’ reports of their parents’ political sophistication and parents’ reports of their political sophistication is used to determine naturally occurring groups, and it is expected that two of these cluster groups will be: adolescents whose parents have a high political sophistication and where the adolescents correctly perceive their parents’ high political sophistication and adolescents whose parents have a high political sophistication but where the adolescents fail to recognize this. The present study also examines the stability of these clusters over one year. With the aim of validating the identified clusters longitudinally, a second cluster analysis is performed on the same sample one year later. Adding to the first hypothesis, if adolescents who correctly perceive their parents’ high political sophistication are more likely than other adolescents to be aware of their parents’ political socialization attempts and be open to their influence cross-sectionally, this might also be true longitudinally. Hence, the predictive utility of the cluster of adolescents who correctly recognize their parents’ high political sophistication for changes in all aspects of their political interactions at home over one year is tested as well.

## Method

### Participants

Participants were recruited from a longitudinal project on young people’s political development, conducted in a city of 137,000 inhabitants in central Sweden (Amnå et al., 2009). Ten schools were strategically chosen to represent the social and demographic characteristics of the city’s adolescents. Both theoretical and vocational educational programs were represented. The city is fairly representative of the country as a whole regarding demographic characteristics and social economic indicators, such as population density, income, unemployment rate and election turnout (Statistics Sweden, 2010).

This study uses a sample of 16-year-old adolescents. The target sample (according to class lists) comprised 1,052 students. Of these, 866 (82%) answered questionnaires at school. Parents answered the questionnaires at home (*N* = 580). Complete responses from parents and adolescents at Time 1 (T1) were available for 505 participants (58% of the analytic sample). The participants’ ages averaged 16.56 years (*SD* = 0.67). Slightly more female than male students participated (*N* = 259, 51%). Parents’ mean age was 45.81 (*SD* = 5.29) years for mothers and 48.47 (*SD* = 6.30) years for fathers. Most parents held a high-school leaving certificate on either a trade/vocational or an academic track (mothers, 37.9% and fathers, 49.6%) or had a university degree (mothers, 54.3% and fathers, 39.7%). Only a few had left school with ten or fewer years of education (mothers, 7.8% and fathers 10.7%). Of the 505 participants with both self-reports and parent reports at T1, 420 (83 %) of the adolescents were followed up one year later at T2.

The data collections took place during regular school hours. Trained test administrators distributed a self-report questionnaire without teachers being present. The students were told that their participation was voluntary. Before visiting the school, the parents of the students were informed by regular mail about the study, including its purpose, procedures, and voluntary nature. The letter to the parents included a pre-paid envelope enabling them to refuse their child’s participation in the project (less than 2% did). The letter also included a questionnaire for the parents with a stamped envelope, which they could use to return the questionnaire to the research team. Each class received a contribution to the class fund of approximately €100 for participation. One of the six regional ethics review boards of the National Ethics Review Board in Sweden approved all the procedures.

### Measures

The measures were developed within the longitudinal project; for most of them, their psychometric properties have been reported earlier.

#### Parents’ political sophistication, adolescent-reported

This indicator comprised two measures of parents’ political interest and knowledge (Stattin & Russo, in press). First, parents’ sociopolitical interest was measured by four questions, “My parents are interested in what is going on in the world” and “My parents keep themselves in touch with the news”, My parents don’t care so much about what is going on in the world (with reversed responses)” and “My parents are not particularly interested in politics or societal issues (with reversed responses)”. The responses ranged from 1 (*doesn’t apply at all*) to 5 (*applies very well*). Cronbach’s alpha was 0.70. Second, parents’ sociopolitical knowledge, as perceived by the adolescents, was measured by a single question “Are your parents knowledgeable about what is going on in Sweden and in the rest of the world?”. It was answered on a scale ranging from 1 (*they have no knowledge of what is going on*) to 5 (*they have very good knowledge of what is going on*).

#### Parents’ political sophistication, parent-reported

This indicator comprised two scales (Stattin et al., 2021). First, parents’ sociopolitical interest was measured with a question on mother’s and father’s sociopolitical interest “How interested are you in politics or societal issues (mother/father)?”. The response scale ranged from 1 (*totally uninterested*) to 5 (*very interested*). The correlation between the responses of the two parents was 0.51, *p* < 0.001. Both responses were aggregated. Second, the extent to which parents followed the news was measured on a scale that aggregated two questions to both the mother and the father. After the stem question: “To what extent does this apply to you?”, the following two alternatives were presented “Can be described as a person who tries to keep informed about what is going on in the world”, and “Follows the news daily to know what other people are talking about”. Mothers and fathers responded on a scale ranging from 1 (*doesn’t apply at all*) to 5 (*applies very well*). Cronbach’s alpha was 0.71.

#### Political interest

The participants were asked two questions (Stattin et al., 2017): “How interested are you in politics?” (a standard measure in the literature; see, for example, the ANES 2008–2009 Panel Study) and “How interested are you in what is going on in society?” They answered on a scale ranging from 1 (*totally uninterested*) to 5 (*very interested*). The correlations between the two variables were 0.59, *p* < 0.001 at T1 and 0.61 at T2. The items were aggregated.

Five measures capturing adolescents’ perceptions of their political interactions with parents and three measures capturing parents’ perceptions of these political interactions were used.

#### Parents’ provision of political information

A three-item scale measured parents’ attempts to provide political information (Stattin et al., 2021): My parents: “Suggest newspapers, books or websites where I can read about politics or societal issues;” “Give me information about activities or organizations in which I can get engaged;” and “Want me to sit down and watch the news on television with them”. The response scale ranged from 1 (*definitely doesn’t apply*) to 5 (*applies very well*). Parents responded to the same three items, slightly reframed (e.g., “We suggest newspapers, books or websites where our child can read about politics or societal issues”), with the same response scales as for the adolescents. Alpha reliability was 0.70 for adolescents and 0.67 for parents. Although these reliabilities are on the low side, the inter-item correlations are adequate: 0.43 and 0.40, respectively.

#### Parents’ attempts to politically influence their adolescents

This measure comprised two questions (Stattin & Russo, in press): “Do your parents try to get you to become more aware of environmental issues?” and “Do your parents try to get you to become more aware of what is going on in the world?”. The same two questions were also posed to parents. The response scales ranged from 1 (*no, never*) to 5 (*yes, almost always*). The correlations between the two adolescent-reported questions were 0.54 at T1 and 0.49 at T2. The correlation for parent reports at T1 was 0.46. The two questions were aggregated.

#### Importance of parents’ views

This indicator comprised the question: “Are your parents’ views on and ways of looking at societal issues important to you?” The adolescents responded on a scale ranging from 1 (*no, not at all*) to 4 (*yes, very*).

#### Susceptibility to parents’ political communication

Adolescents answered three questions (Stattin & Russo, in press) about whether their parents… “… talk about politics and societal issues in a way that makes them fun and interesting”, “… talk about things that happen in the world and in society in such a way that I become curious and want to know more”, and “… tell me about the news they have heard on TV or read about in a way that evokes strong feelings in me”. The response scale ranged from 1 (*definitely does not apply*) to 5 (*applies very well*). Cronbach’s alpha was 0.85 at T1 and 0.86 at T2.

#### Political discussions

Perceptions of the frequency of political discussions at home (Stattin et al., 2021) were examined. Participants were asked how often they discussed politics with parents: “What you have heard on the news about what is going on in Sweden and around the world”, and “Politics or societal issues”. The response scales ranged from 1 (*never*) to 4 (*very often*). The correlation between the two question responses was 0.53 at T1 and 0.59 at T2. They were aggregated at both T1 and T2. Parents responded on the same questions. The correlation between the two items was 0.44.

In order to avoid problems due to conducting multiple comparisons, which increases Type 1 errors, measures based on adolescents’ reports about their political interactions with parents (i.e., parents’ provision of political information, parents’ attempts to politically influence their adolescents, importance of parents’ views, susceptibility to parents’ political communication, and political discussions) were factor analyzed. One factor emerged with factor loadings ranging from 0.71 to 0.82. At T2, the same measures again yielded one factor with factor loadings ranging from 0.72 to 0.83. The three measures based on parents’ reports about their political interactions with their children (i.e., providing political information, attempts to politically influence their adolescents, and political discussions) also yielded one factor, with factor loadings ranging from 0.76 to 0.82. These three factor scores were saved and used in the main analyses. A Supplementary Materials Appendix reports the analyses for the original measures.

#### Control variables

An important question is whether the study findings can be generalized across individual, social, and educational characteristics. Therefore, gender, immigrant status, and parental level of education were included as control variables in all main analyses. When significant differences appear, they are reported in the running text.

Gender was coded 1 = *male*, 2 = *female*. Immigrant status was a dichotomous measure, differentiating between participants both of whose parents were born outside the Nordic countries (coded 2) and other participants (coded 1). The question about fathers’ and mothers’ education was answered on a 5-point scale ranging from 1 (*less than 9 years of education*) to 5 (*university college/university education*). Mothers’ and fathers’ scores on educational level were averaged.

### Plan of Analysis

Adolescent- and parent-reported measures of political sophistication were factor analyzed (principal factor analysis with promax rotation). The resulting indicators of “Parents’ political sophistication” and “Adolescents’ perceptions of parents’ political sophistication” were then cluster analyzed. First, a hierarchical cluster analysis (Ward’s method) was applied to identify the number of clusters for these two measures. The lower explanatory limit was set at 67% of the total error sum of squares for the number of clusters selected (Bergman et al., 2003). Second, as recommended by Kinder et al. (1991), with knowledge of the number of clusters, a non-hierarchical cluster analysis, K-means clustering, was used to arrive at the final cluster solution. Regular ANOVAs were then used to examine differences among the cluster groups at T1 for all measures of adolescents’ political interactions with their parents and of adolescents’ own political interest, controlling for gender, immigrant status, and parents’ educational level.

With the aim of validating the clusters longitudinally, the clusters obtained at T1 were cross-tabulated with those obtained one year later in the sample, at T2. The EXACON program for single-cell contingency table analysis (Bergman & El-Khouri, 1987), with a Bonferroni adjusted *p*-value of 0.05, was used to determine which specific cells occurred more often (a Type) and less often (an Antitype) than expected by chance.

In a final step, regression analysis was used to predict adolescents’ perceptions of political interactions with their parents at T2, controlling for their political interactions with parents at T1. Here, the categorical variable, cluster grouping at T1, was dummy coded with the cluster “Parents have low political sophistication and adolescents perceive their parents to have low political sophistication” as the reference group.

### Attrition Analyses

One of two types of attrition analysis was performed depending on whether the question was cross-sectional or longitudinal. First, concerning the cross-sectional analyses, logistic regression was used to compare the T1 participants whose parents responded (*N* = 505) with the T1 participants whose parents did not respond (*N* = 361). This analysis included all parent and adolescent reports at T1 and the covariates. Significant differences were obtained for parents’ political sophistication (OR = 1.42, *p* = 0.045), adolescents’ reports of parents’ political sophistication (OR = 1.22, *p* = 0.036), and immigrant status (OR = 2.25, *p* < 0.001). Participants whose parents responded showed higher-level parent and adolescent reports of parents’ political sophistication, and formed a group with fewer individuals of immigrant descent. Nagelkerke *R*^*2*^ was 0.06. Second, in the longitudinal analyses, where adolescent reports on their political interactions with their parents at T2 were used, comparisons were made between participants with self and parent reports at T1 and with self-report data at T2 (*N* = 420) and those who lacked T2 data (*N* = 95). This analysis included all parent and adolescent reports at T1 and the covariates. There was one significant difference. Participants with data at both T1 and T2 reported greater attempts by parents to politically influence them than the other participants (OR = 1.50, *p* = 0.012). Nagelkerke *R*^*2*^ was 0.04. In view of low Nagelkerke *R*^*2*^ coefficients, and given that the differences in terms of significance when converted to Cohen’s *d* can be regarded as small, the bias due to data attrition can be considered to be low.

## Results

### Profiles of Parents’ Political Sophistication

Responses to the questions about parents’ political interest and political knowledge, posed to both parents and adolescents, were factor analyzed. As shown in Table 1, two factors emerged at T1, labeled “Parents’ political sophistication” and “Adolescents’ perceptions of parents’ political sophistication”. The resulting factor scores were used in the following cluster analysis. First, in line with earlier recommendations (Bergman et al., 2003), a hierarchical cluster analysis identified five clusters which explained 71 percent of the total error sums of squares. A subsequent non-hierarchical cluster analysis produced the clusters shown in Table 2. As expected, there was a group of adolescents with highly politically sophisticated parents who correctly recognized their parents’ high political sophistication, from here on labeled the HH adolescents (*n* = 133, 26%), and a group of adolescents with highly politically sophisticated parents who failed to recognize their parents’ high sophistication (*n* = 109, 22%), from here on labeled the HL adolescents. In addition, three clusters emerged where both parents and adolescents reported low or moderate levels of parental political sophistication (LL: both parents and adolescents report that parents have low political sophistication; LM: parents report low political sophistication and adolescents report that parents have medium political sophistications; MM: both parents and adolescents report that parents have medium political sophistication). It should be noted that the correlation between the parents’ political sophistication and the adolescents’ perceptions of their parents’ political sophistication was 0.29, *p* < 0.001, for the whole sample. The same correlation, excluding the HH adolescents, was −0.04, *p* = 0.437. Hence, it is only by chance that the adolescents in the clusters other than the HH correctly perceive their parents’ political sophistication as high or low.

**Table 1:** Factor analysis of measures of parents’ political interest and knowledge and adolescents’ perceptions of parents’ political interest and knowledge

	Parents’ sophistication	Adolescents’ perceptions of parents’ sophistication
Parent reports: Politically interested	0.65	0.04
Parent reports: Follow the news	0.60	−0.02
Adolescent reports: Parents are interested	0.03	0.73
Adolescent reports: Parents are knowledgeable	−0.04	0.67

**Table 2:** A cluster analysis of the measures of parents’ political sophistication and adolescents’ perceptions of their parents’ political sophistication at T1 and a cross-validation at T2 for all adolescent-parents dyads at this point in time (N = 362).

Parents (T1):	Low	Low	Medium	High	High
Adolescents (T1):	Low	Medium	Medium	Low	High
Label:	LL	LM	MM	HL	HH
Parents’ political sophistication	−0.74	−1.86	−0.42	0.80	0.79
Adolescents’ perceptions of parents’ sophistication	−1.66	−0.30	0.12	−0.59	1.13
N (%)	55 (11)	45 (10)	163 (32)	109 (22)	133 (26)
% females^1^	51	49	54	44	55
% immigrants^2^	13	7	13	10	15
Parents’ education (z-scores)^3^	−0.33^a^	−0.42^a^	−0.13^a^	0.21^b^	0.35^b^
Parents (T2):	Low	Low	High	High	
Adolescents (T2):	Low	High	Low	High	
Label:	LL	LH	HL	HH	
Parents’ political sophistication	−0.98	−0.50	0.78	0.95	
Adolescents’ perceptions of parents’ sophistication	−0.75	0.79	−0.60	1.17	
N (%)	112 (31)	77 (21)	101 (28)	72 (20)	
% females^4^	44	56	52	54	
% immigrants^5^	9	8	11	12	
Parents’ education (z-scores)^6^	−0.29^a^	0.27^b^	0.12^b^	0.29^b^	

*Low values (L)* lowest value through −0.50, *Medium values (M)* −0.49 through 0.49, *High values (H)* 0.50 through highest value

^1^Chi^2^ (4 df) = 3.57, *p* = 0.468

^2^Chi^2^ (4 df) = 2.38, *p* = 0.667

^3^F (4, 499) = 10.22, *p* < 0.001

^4^Chi^2^ (3 df) = 2.85, *p* = 0.415

^5^Chi^2^ (4 df) = 0.96, *p* = 0.810

^6^F (3, 292) = 7.16, *p* < 0.001

For parents’ education, across rows, the superscript letters represent significant differences (*p* < 0.05) between clusters in SNK post-hoc tests

Table 2 reports on the clusters in the sample that emerged one year later. Five clusters were identified, explaining 73 percent of the total variance. Because two of them contained adolescents who correctly identified their parents’ low political sophistication, and one of them was more extreme than the other, a decision was made to report a four-cluster solution (65% of the total variance). As shown in the lower part of Table 2, the HH and the HL adolescents again appeared as separate clusters (*n* = 72; 20% and *n* = 101; 28%, respectively), in addition to a cluster where adolescents reported that their parents had high political sophistication, but where the parents, to the contrary, reported low political sophistication, and also a cluster where the adolescents correctly identified their parents’ low political sophistication. Overall, there is confirmation of the presence of groups of adolescents who correctly recognize their parents’ high political sophistication and of those who fail to recognize their parents’ high political sophistication. Concerning the covariates reported in Table 2, at both T1 and T2, no significant gender differences or differences with regard to immigrant status were found between the clusters. However, there were significant differences for parents’ level of education, which was highest among the HL and the HH adolescents at T1, and highest among the LH, HL, and HH adolescents at T2.

A cross-tabulation of the T1 and T2 clusters is reported in Table 3. This analysis showed a high stability of the T1 clusters. The contingency coefficient was 0.59, *p* < 0.001. Apart from the “types”, where the T1 clusters appeared more often than expected by chance at T2 (indicating high stability over time for these specific cells), it is noteworthy that the HL adolescents at T1 were less likely than expected by chance to appear in the HH cluster at T2, and that the HH adolescents at T1 were less likely than expected by chance to appear in the HL cluster at T2. In essence, the participants in these two clusters seem to show opposite developmental trends over time.

**Table 3:** Cross-tabulation of the clusters between T1 and T2

		T2			
		Parents: low Adolescents: low	Parents: low Adolescents: high	Parents: high Adolescents: low	Parents: high Adolescents: high
T1	Parents: low Adolescents: low	15^t^	0	9	0
	Parents: low Adolescents: medium	16^t^	3	3	0
	Parents: medium Adolescents: medium	33	25	19	12
	Parents: high Adolescents: low	13	10	36^t^	5^a^
	Parents: high Adolescents: high	2^a^	18	8^a^	41^t^

The first figure is parents’ political sophistication and the second is adolescents’ perceptions of parents’ political sophistication

^a^Antitype: the cell frequency is smaller than expected by change

^t^Type: the cell frequency is larger than expected by chance

### Parents’ Socialization Attempts and Adolescents’ Perceptions of the Political Interactions With Parents

According to the first study hypothesis, the HH adolescents, in particular, will perceive higher levels of political interactions with their parents (i.e., have frequent political discussions with their parents, be aware of their parents’ political socialization attempts, be open to their parents’ political communication, and recognize having joint political discussions). Hence, a regular ANOVA was used to compare the adolescents in the five cluster groups on the factor measure that captured adolescents’ reports on these political interactions (Table 4). In line with the hypothesis, the HH adolescents had significantly higher means on this measure than adolescents from other clusters. The effect size was high (eta^2^ = 0.19). Significantly higher means for the HH adolescents than for adolescents from other clusters were also found when examining all original indicators one by one (for detailed information, see Supplementary Materials, Table S1).

**Table 4:** Differences between the five cluster groups for adolescent- and parent-reported measures of their political interactions (factor scores) and the adolescents’ political interest

	Cluster group:							
	1	2	3	4	5	*F*	*p*	eta^2^
Parents:	Low	Low	Medium	High	High			
Adolescents:	Low	Medium	Medium	Low	High			
Label:	LL	LM	MM	HL	HH			
Political interactions (A)	−0.83^a^	−0.29^b^	0.05^c^	−0.22^b,c^	0.69^d^	30.60	<0.001	0.19
Political interactions (P)	−0.50^a,b^	−0.71^a^	−0.21^b^	0.35^c^	0.51^d^	23.81	<0.001	0.16
Political interest (A)	−0.40^a^	−0.23^a,b^	0.08^b^	−0.05^a,b^	0.52^c^	8.90	<0.001	0.07

All measures in the table are standardized. Across rows, superscript letters represent significant differences (*p* < 0.05) between clusters in SNK post-hoc tests

*A* Adolescent reports, *P* parent reports

Turning to parental reports of political interactions (i.e., having regular discussions about political issues, influence and giving political support to their children) higher levels were expected in both the HL and the HH cluster groups. As shown in Table 4, the factor measure capturing parental reports, confirms this. Again, the effect size was high (eta^2^ = 0.16). Significantly higher means for both the HL and the HH adolescents than for adolescents in the other cluster groups were also found when examining the three original indicators one by one (for detailed information, see Supplementary Materials, Table S1). Overall, when focusing on parents’ report, the findings in Table 4 suggest that politically sophisticated parents try to politically socialize their children more than other parents. However, among the adolescents in these families, it is mainly the ones who are able to correctly recognize their parents’ high political sophistication who identify their parents’ political socialization attempts and are open to parents’ political communication. The findings support the first hypothesis. Concerning covariate effects, parents’ education was found to be significant at the 0.001 level for adolescent reports of political interactions and at 0.002 for parental reports of political interactions. Immigrant status had a significant effect at the 0.001 and 0.021 level for both political interaction measures. Political interactions were higher among participants with highly educated parents and among participants with at least one parent born in Sweden.

### Parents’ Socialization Attempts and Adolescents’ Political Interest

The second hypothesis focuses on the role of adolescents’ political interest. The ANOVA results reported at the bottom of Table 4 confirm that the HH adolescents had significantly higher levels of political interest than the other cluster groups of adolescents. The effect size was medium (eta^2^ = 0.07). Parents’ education was significant at the 0.012 level and immigrant status was significant at the 0.001 level. Political interest was higher among participants with highly educated parents and among participants with at least one parent born in Sweden.

### Across-Time Effects of Parents’ Socialization Attempts

If the ability to correctly recognize parents’ high political sophistication is associated with features that are contemporaneously beneficial for the political development of adolescents, a critical question is whether this ability can also be an asset for adolescents’ future political interactions with their parents. This is an extension of the first hypothesis. Here, the factor covering adolescents’ perceptions of political interactions with their parents at T2 was the dependent variable.

A regression analysis was performed where the five clusters were reflected in four dummy-coded indicators, with the LL cluster as the reference category. A positive significant regression coefficient indicates that adolescents’ perceptions of political interactions with their parents at T2 is significantly higher for the particular cluster group than for the reference group after controlling for the stability of the outcome variable and covariate effects. The results of these analyses are reported in Table 5. As shown in the table, three of the clusters, MM, HL, and HH, had significantly higher means than the reference group. The strongest effect was found for the HH cluster. No significant effects were found for the covariates. Results from additional analyses, which accounted for the original five measures, are presented in the Supplementary Materials, Table S2. The findings show that adolescents in the HH cluster differed significantly at high levels of significance from the reference group for all individual measures of political interactions. Thus, for changes over one year in the perception of parents’ political interactions the cluster in which adolescents correctly recognized their parents’ high political sophistication was a significant predictor. There were two significant effects for the HL cluster and three for the MM cluster, but the significance was only at the 0.05 level. Consequently, belonging to the HH cluster group appears generally beneficial for these adolescents’ future political interactions with their parents.

**Table 5:** Predictions of adolescents’ perceptions of political interactions with their parents at T2 from their cluster membership at T1

	beta	*SE*	*t*	*p*
Variable at T1	0.65	0.04	16.87	<0.001
Low-Medium	0.07	0.04	1.45	0.093
Medium-Medium	0.17	0.06	2.60	0.005
High-Low	0.15	0.05	2.46	0.007
High-High	0.25	0.06	3.95	<0.001
*R*^*2*^	0.55			

Reference category is Cluster 1: Low level of parents’ political sophistication and low level of adolescents’ perception of parents’ political sophistication

## Discussion

Low congruence in political interest, sociopolitical values, and attitudes between parents and adolescents has been found in research (Jennings et al., 2009; Neundorf et al., 2013; Oswald & Schmid, 2006). Moreover, adolescents seem to have limited insight into their parents’ values and political interest (Stattin & Kim, 2018 Stattin et al., 2021). Further, with few exceptions (Jennings et al., 2009), political socialization studies have not shown many examples of conditions that substantially increase the likelihood that adolescents adopt their parents’ political interests and views. Employing a person-centered approach, the present study shows that at least some adolescents are open to their parents’ political socialization efforts.

Different theoretical models converge on the assumption that, if parents’ attitudes are to influence their children’s attitudes, the children have to have an accurate perception of parental values (Grusec & Goodnow, 1994; Ojeda & Hatemi, 2015; Tedin, 1974). This study reformulates this perceptual accuracy argument and makes the broader assumption that adolescents who accurately recognize that their parents have high political sophistication—high political interest and high political knowledge—will be particularly likely to be open to their parents’ political socialization attempts and perceive that they often talk to their parents about political issues.

An analysis that simultaneously clustered adolescents’ perceptions of their parents’ political sophistication and parents’ reports of their own political sophistication yielded five cluster groups. The two key clusters were a group of adolescents who correctly perceived that their parents were highly politically sophisticated and a group of adolescents who failed to perceive their parents’ high political sophistication. In agreement with the first study hypothesis, adolescents who correctly perceived their parents’ high political sophistication could identify their parents’ attempts to provide political information and recognize their parents’ attempts to influence them politically. Moreover, these youth were more open to their parents’ political communication and viewed their parents’ opinions as important than adolescents in all the other cluster groups. In short, they seemed to see their parents as political role models. Finally, this group of adolescents also perceived that they had joint discussions about politics and society more often than adolescents in the other cluster groups. The adolescents’ correct perceptions of their parents’ high political sophistication and greater openness to parents’ political socialization, seem to provide one explanation for why some families are genuinely politicized whereas others are not. Altogether, these findings support the first hypothesis of the current study that adolescents who accurately recognize their parents’ high political sophistication recognize their parents’ efforts to politically socialize them and have an openness to parents’ attempts at political socialization.

A longitudinal validation of the clusters was performed one year later. The results showed that both adolescents who made correct and incorrect judgements about their parents’ high political sophistication tended to remain in their respective clusters over time. In addition, if adolescents in the cluster who failed to recognize their parents’ high political sophistication changed over the year, they were not likely to move to the cluster where adolescents correctly perceived their parents’ high political sophistication. This was also the case the other way round. This indicates that the political developments of these two clusters of adolescents tend to proceed quite separately from each other. Notably, correctly perceiving one’s parents as highly politically sophisticated is not for everyone. It is an insight that only applies to a minority of young people: in the current study, 26 percent at the age of 16, and 20 percent one year later.

The second hypothesis assumed that adolescents need to have some degree of political interest to be able to correctly recognize their parents’ political interest and knowledge, to understand that their parents try to support their political development, and to recognize that they have joint political discussions. Ample support was found for this hypothesis. Potentially, these adolescents’ perceptions of their parents’ political interest and knowledge may not be a “neutral” or value-free perception but may partly depend on the adolescents’ own political interest. This suggests that an understanding of the political interactions that occur in these families may need to encompass adolescents’ political agency.

Regression analyses were used to examine whether the five different cluster groups could predict all key measures of the adolescents’ reports of political interactions with their parents one year later. These longitudinal analyses revealed that positive changes over time primarily occurred in the cluster of adolescents who accurately recognized their parents’ political sophistication. It seems that the adolescents in this cluster have a head start in their political development.

The present study also accounted for parents’ perspectives. The results revealed that in the two cluster groups containing highly politically sophisticated parents, parents provided their adolescent children with political support, talked with their children about political and societal issues, and tried to influence their children’s views more than other parents. These parents also had a higher educational level than less politically sophisticated parents. There were no significant differences between the groups of adolescents who correctly perceived or who failed to perceive their parents’ high political sophistication. Apparently, highly sophisticated parents are keen to attempt to politically socialize their adolescent children rather independently of their adolescents’ ability to recognize their high political skills. In a sense, this is perhaps what might be expected of politically sophisticated parents who want to support their adolescents’ political development. Similar findings typically appear in the political socialization literature (e.g., Jennings et al., 2009), but, as was shown above, the similarities between these two groups of adolescents end here. It is only among the adolescents with correct perceptions of their parents’ high political sophistication that an openness to their parents’ political socialization can be witnessed.

The present study provides a counter image to that of previous attempts to specify the conditions for a successful transmission of parents’ values and attitudes. The study by Jennings et al. (2009) offers the best-known explanation for why adolescents’ values and attitudes are similar to those of their parents. Building on a social learning perspective, Jennings and coworkers examined which aspects of parents’ political socialization attempts tend to make adolescents ready to adopt their parents’ views. They found that parents needed to express their attitudes consistently over time in order for transmission of their attitudes and values to take place. The value of the present study is that it takes the adolescents’ perspectives and specifies when greater openness to their parents’ political socialization attempts can be expected. These two perspectives, those of parents and adolescents, do not necessarily contradict each other. Seen in juxtaposition, they are likely to complement each other and offer new avenues for future studies to provide a fuller picture of the successes and failures of parents’ attempts to politically socialize their children.

The main strength of the present study is that it applies and largely confirms a model of how adolescents’ perceptions of their parents’ high political interest and knowledge are related to their recognition of parents’ political support and influence and acceptance of parents’ political messages. The study findings generalize across key individual, social, and educational characteristics, such as gender, immigrant status, and parents’ educational level. Another strength of the study lies in its multi-informant design, with both parent and adolescent reports.

The main limitation of the study is that it does not offer causal conclusions. The study hypothesis is that an accurate perception of one’s parents’ high political sophistication benefits adolescents in recognizing their parents’ political support and influence and makes them susceptible to their parents’ political communication. The reported longitudinal analyses support such an interpretation. The directions of effects are theoretically grounded, but this is no guarantee that the causal sequences also can be in the opposite direction. In particular, adolescents’ own political interest may color their perceptions of having political discussions with their parents and the political support they receive from parents, as well as being affected by these discussions and support (Stattin et al., 2021).

The study was conducted at a specific point in time and in a specific geographical milieu. The sample can be regarded as representative of adolescents of the same age in Sweden at the time the study was conducted. However, there is no reason to believe that the same clusters found in this Swedish sample will necessarily turn up in samples from other countries with different characteristics. There may already be data sets in other countries that cover the key features of this study: measures of parents’ political interest and knowledge and of their children’s perceptions of their parents’ political interest and knowledge. If this is the case, it would be possible to quickly conduct a cross-validation of the results reported here.

## Conclusion

The lack of congruence between parents’ and adolescents’ sociopolitical values and attitudes, reports about political discussions, and political interest, prompted this study. The current study examined the conditions that make adolescents open to their parents’ political socialization attempts. Cluster analyses of adolescents’ perceptions of their parents’ political sophistication and parents’ reports of their political sophistication give evidence that at least some adolescents have an openness to their parents’ political socialization efforts. These are adolescents who correctly perceive their parents’ high political sophistication. The study has three take-home messages. First, a correct perception on the part of adolescents of their parents’ high political sophistication paves the way for recognizing parents’ political socialization attempts and an openness to parents’ political communication. Such openness indicates that these adolescents are more ready to accept their parents as political role models than other adolescents. Second, there is longitudinal evidence that the ability to make a correct prediction of their parents’ high political sophistication is beneficial for these adolescents’ future political interactions with their parents. Third, according to parental reports, politically sophisticated parents often talk with their adolescents about political and societal issues, give their adolescents political support, and try to influence their adolescents’ views to a greater extent than other parents. This should be expected; politically sophisticated parents want to support their adolescents’ political development (Jennings et al., 2009). But it is primarily the adolescents who perceive the high political sophistication of these parents who are open to their parents’ political socialization attempts. These three conclusions open up new paths for future studies aimed at specifying the conditions for the successful intergenerational transmission of values.

## Supplementary Information

Supplementary Information
