# Supplementary material for: Why Some Adolescents Are Open To Their Parents’ Political Communication
Source: J Youth Adolesc. 2022 Jul 8;51(11):2235–47. doi: 10.1007/s10964-022-01653-x (PMC9508036; doi:10.1007/s10964-022-01653-x)
Supplement: Supplementary file 1 — Supplementary Information [file 10964_2022_1653_MOESM1_ESM.docx]

**Table S1**. Differences between the five cluster groups for adolescent- and parent-reported measures of their political interactions (item level).

___________________________________________________________________________________________________________________________

Cluster group:

1 2 3 4 5 *F p* eta^2^

Parents: Low Low Medium High High

Adolescents: Low Medium Medium Low High

Label: LL LM MM HL HH

___________________________________________________________________________________________________________________________

Adolescent reports:

Joint political discussions -0.77^a^ -0.10^b^ 0.04^b^ -0.19^b^ 0.60^c^ 22.93 <.001 0.18

Parents’ provision of information -0.42^a^ -0.33^a^ -0.05^a^ -0.12^a^ 0.46^b^ 9.82 <.001 0.07

Parents’ attempts to influence -0.66^a^ -0.17^b^ 0.16^c^ -0.19^b^ 0.48^d^ 16.88 <.001 0.12

Parents’ views are important -0.66^a^ -0.14^b^ 0.05^b^ -0.22^b^ 0.44^c^ 12.01 <.001 0.09

Susceptible to parents’ communication -0.68^a^ -0.34^b^ -0.01^c^ -0.17^bc^ 0.65^d^ 22.18 <.001 0.15

Parent reports:

Joint political discussions -0.42^b^ -0.86^a^ -0.14^c^ 0.30^d^ 0.49^d^ 21.66 <.001 0.15

Parents provide information -0.32^a^ -0.39^a^ -0.26^a^ 0.28^b^ 0.38^b^ 13.08 <.001 0.10

Parents attempt to influence -0.45^a^ -0.46^a^ -0.14^a^ 0.23^b^ 0.35^b^ 10.46 <.001 0.08

____________________________________________________________________________________________________________________________

*Note*. All measures in the table are standardized. Across rows, superscripts represent significant differences (p < .05) between clusters in SNK post-hoc tests.

**Table S2**. Predictions of adolescents’ perceptions of the political interactions with their parents at T2 from their cluster membership at T1. (Item level).

______________________________________________________________________________________________________

beta *SE t p* beta *SE t p*

_______________________________________________________________________________________________________

*Parents’ provision of political information: Parents’ attempt to influence:*

Variable at T1 .48 .04 11.02 <.001 .54 .04 13.13 <.001

Low-Medium -.01 .05 -0.16 .877 .06 .05 1.18 .240

Medium-Medium .10 .07 1.42 .155 .11 .07 1.62 .107

High-Low .06 .06 0.98 .329 .13 .06 2.10 .037

High-High .17 .07 2.58 .010 .25 .07 3.85 <.001

*R^2^* .33 .43

*Perception that parents’ views are important: Susceptible to parents’ communication:*

Variable at T1 .43 .05 9.32 <.001 .53 .04 12.14 <.001

Low-Medium .08 .06 1.44 .151 .10 .05 1.96 .051

Medium-Medium .19 .07 2.54 .011 .13 .06 2.01 .046

High-Low .13 .07 1.91 .057 .10 .06 1.59 .113

High-High .20 .07 2.72 .007 .28 .07 4.27 <.001

*R^2^* .25 .40

*Frequency of political discussions:*

Variable at T1 .53 .04 12.39 <.001

Low-Medium .02 .05 0.43 .665

Medium-Medium .16 .07 2.48 .013

High-Low .13 .06 2.22 .027

High-High .27 .07 4.07 <.001

*R^2^* .39

_______________________________________________________________________________________________________

Note. Reference category is Cluster 1: Low level of parents’ political sophistication and Low level of adolescents' perceptions of parents’ political sophistication.
